# A Semiautomated ChIP-Seq Procedure for Large-scale Epigenetic Studies

**DOI:** 10.3791/61617

**Published:** 2020-08-13

**Authors:** Justin Cayford, Sara Herrera-da la Mata, Benjamin Joachim Schmiedel, Vivek Chandra, Pandurangan Vijayanad, Grégory Seumois

**Affiliations:** 1 Division of Vaccine Discovery, La Jolla Institute for Immunology

## Abstract

Chromatin immunoprecipitation followed by sequencing (ChIP-Seq) is a powerful and widely used approach to profile chromatin DNA associated with specific histone modifications, such as H3K27ac, to help identify cis-regulatory DNA elements. The manual process to complete a ChIP-Seq is labor intensive, technically challenging, and often requires large-cell numbers (>100,000 cells). The method described here helps to overcome those challenges. A complete semiautomated, microscaled H3K27ac ChIP-Seq procedure including cell fixation, chromatin shearing, immunoprecipitation, and sequencing library preparation, for batch of 48 samples for cell number inputs less than 100,000 cells is described in detail. The semiautonomous platform reduces technical variability, improves signal-to-noise ratios, and drastically reduces labor. The system can thereby reduce costs by allowing for reduced reaction volumes, limiting the number of expensive reagents such as enzymes, magnetic beads, antibodies, and hands-on time required. These improvements to the ChIP-Seq method suit perfectly for large-scale epigenetic studies of clinical samples with limited cell numbers in a highly reproducible manner.

## Introduction

The wide use of ChIP-Seq assays for determining fragments of DNA associated with specific histone modifications is in part due to its ability to identify cis-regulatory DNA elements, including active enhancers, promoters, silencers, heterochromatin, and others^1, 2, 3, 4^. Identification of non-coding regulatory regions across the genome has shown valuable insight to better understand gene regulation in health and diseases^4^. Previous work from the lab has used ChIP-Seq to show that cis-regulatory elements can play important roles in different cell types^5^. Transcription factor (TF) ChIP assays has been utilized to show disease associated risk single-nucleotide polymorphisms^6^.

The use of ChIP-Seq with human clinical samples is challenging, mainly due to the limitation of cell numbers or the desired tissue sample. As a result, there has been a concerted effort in the field to improve and microscale these techniques and as a result, several assays have emerged, such as CUT&TAG^5, 7, 8, 9, 10, 11, 12^. This assay utilizes a transposase to tagment and isolate genomic regions bound by a specific antibody^9^. This technique has been able to reduce the cell numbers down to 1,000s and in some cases to a single-cell, however, the use of this technique in translational research and clinical set-up has shown limitations due to the requirements of using live cells for this method^9, 12^. The live cell requirement makes clinical samples logistically difficult to handle and can introduce batch effects if the samples are not processed at the same time. Others have optimized microscaled techniques for formaldehyde-fixed cells, including the development of ChIPmentation^11^, which is adapted here in a high-throughput manner. The use of fixed cells allows samples to be stored until collection and subsequent processing of all samples together to minimize batch effects.

Here, a semiautomated microscaled ChIP-Seq assay is described which reduces experimental hands-on time to profile histone modifications^10^. The semiautomated method allows for high-throughput ChIP-Seq assays, allowing for up to 48 samples to be fully processed and ready for sequencing in as little as 5 days, for as few as 10,000 cells per sample using a ChIP liquid-handler. The handler completes the immunoprecipitation (IP) and subsequent washes in an autonomous manner, which helps to reduce variability between samples. The semiautomated method lowers both the hands-on time by over 15 h for 48 samples and the technical variability, enabling large-scale epigenetic studies to be conducted in a reproducible and rapid manner for either primary or cultured cells. The protocol explains the process from start to finish for high quality ChIP-Seq. If the specific machines are not available, the protocol will still be a useful resource to set up and trouble-shoot ChIP-Seq experiments manually.

The assay was performed with three different primary human immune cell types and one cultured cell line (HUT78 – ATCC: TIB-161). For clarity, the protocol has been divided into seven sections: cell fixation, chromatin shearing via sonication, automated chromatin immunoprecipitation, library preparation by DNA fragment tagmentation, library amplification, library purification, followed by DNA quantification. For buffer recipes please refer to Supplementary Table 1.

## Protocol

The Institutional Review Board (IRB) of the La Jolla Institute for Allergy and Immunology (LJI; IRB protocol no. SGE-121–0714) approved the study. Healthy volunteers were recruited and provided leukapheresis samples after written informed consent.

### 1. Cell fixation

1. Bring the cell suspension concentration to 1 to 2 × 10^6^ cells/mL with complete cell culture medium in a 15 mL tube (<10 mL of suspension) or 50 mL tube (10–30 mL of cells). If <1 × 10^6^ cells, use 0.5 mL of the medium in a 1.5 mL tube.
2. Gently vortex the cell suspension, add 10x cell fixation buffer dropwise (1:10; vol:vol) and rotate at a low speed for 10 min at room temperature (RT).
3. Stop the reaction by gently vortexing and add 2.5 M glycine in the ratio of 1:20 (vol:vol). Invert the tubes a few times and incubate on ice for at least 5 min.
4. Perform the remaining steps at 4 °C or on ice. Spin the tubes at 800 *x g* for 5 min at 4 °C and discard the supernatant.
5. Resuspend the pellet gently with 5 mL of ice-cold 1x PBS and incubate for 2 min on ice.
6. Repeat 1.4 and 1.5 with 1 mL of ice-cold 1x PBS and transfer the sample to a precooled 1.5 mL tube (labeled for long-term storage). If applicable, preparation of aliquots is recommended.
7. Spin the tubes at 1,200 *x g* at 4 °C and remove as much of the supernatant as possible without disturbing the cell pellet. Snap freeze the pellet in liquid nitrogen. Store at −80 °C.
  CAUTION: Take appropriate protection when handling liquid nitrogen.

### 2. Chromatin shearing

NOTE: This protocol is optimized for the chromatin shearing of pellets with 0.3 to 3 × 10^6^ cells in 0.65 mL low binding tubes.

1. Remove the samples tube with frozen cell pellets from −80 °C and store on dry ice to avoid any thawing of the pellet prior to adding the lysis buffer. This step is critical.
2. Add 70 μL of fresh, RT complete lysis buffer to the pellet and keep at RT for 1 min.
3. Resuspend the pellet for 1 min without the introduction of any bubbles and then incubate the cell suspension at RT for 1 min before putting the sample on ice.
4. Transfer the resuspended pellet into a 0.65 mL low binding tube and keep on ice.
  NOTE: To obtain reproducible sonication, pre-warm the sonicator by running it with only blank tubes for 3–6 cycles prior to sonicating the samples.
5. Place the samples into the tube holder of the sonicator and fill any gaps with balance tubes filled with 70 μL of water. Leave the samples in the water bath for about 1 min before starting the sonication.
6. Perform sonication for *x cycles* (depending on the cell type) with 16 s ON / 32 s OFF per cycle.
  NOTE: This step will require validation experiments to determine the optimal number of cycles for efficient sonication.
7. After every 3 cycles, remove the samples from the sonicator, gently vortex and pulse spin the tubes before putting them back into the holder. Ensure there are no small droplets on the outside of the tube as that can cause bubble formation.
8. After completing the necessary cycles, spin samples at >14,000 *x g* for 15 min at 4 °C. Transfer the supernatant to a new, pre-cooled, low-binding 0.65 mL low binding tube and keep on ice.
9. Decrosslink a fraction of the sonicated samples to check for the sonication efficiency.
  1. Transfer 1–7 μL of the supernatant (equivalent to about 250 ng of sheared chromatin) to a 0.2 mL PCR tube and make the volume up to 10 μL with short-term lysis buffer at RT.
  2. Add 1 μL RNase A and incubate for 30 min at 37 °C at 800 rpm, then add 1 μL proteinase K.
  3. Incubate for 2 h at 55 °C with shaking at 1,000 rpm.
10. Remove 2 μL of the decrosslinked sample for DNA quantification using fluorescent quantification assay^10^ (a spectrophotometer is not recommended as soap and degraded protein can produce bias in the results).
11. Run the remaining sample on a 1.2% agarose gel for 1 h at 70 V. Stain with nucleic acid dye (1:20,000) and read the gel using a UV transilluminator.
12. Prepare chromatin stock aliquots for storage (dilute the sample to 25 ng/μL in 20 μL with complete lysis buffer). Store all sheared chromatin at −80 °C.

### 3. Automated ChIP-Seq for histone modification

NOTE: This protocol is designed to run on a ChIP liquid-handler. Although the system can use customized buffers, all buffers are provided with the ChIP kit. The ChIP strips with 8 tubes used in this section are specific to the ChIP liquid-handler.

1. Transfer 16 sample aliquots with 500 ng of sheared chromatin in 20 μL from −80 °C and place them on ice to slowly thaw the chromatin. Once thawed fully, vortex briefly and pulse-spin.
2. Preparation of chromatin
  1. Pipette 100 μL of tC1 buffer supplemented with 1x protease inhibitor and 20 mM sodium butyrate (complete tC1 buffer) into two ChIP 8-tube strips.
  2. Transfer 20 μL of each chromatin sample into an appropriate tube of the ChIP 8-tube strips containing the 100 μL of complete tC1 buffer. Wash the chromatin tubes by adding 80 μL complete tC1 buffer to the chromatin tubes and then transfer back into the appropriate tube of the ChIP 8-tube strips for a final volume of 200 μL.
3. Preparation of the antibody
  1. Calculate the volume of antibody such that 0.5 μg of antibody is in each tube.
    *Volume of antibody* = (*number of samples x antibody per reaction*) / *antibody concentration*
  2. Add the calculated amount of antibody into 500 μL of tBW1 buffer. Quickly vortex and pulse-spin.
  3. Pipette 70 μL of tBW1 into each of the two ChIP 8-tube strips and add 30 μL of the antibody + tBW1 to each of the tubes. This will bring the total volume in each of the tubes to 100 μL.
4. Preparation of the magnetic bead
  1. Vortex the protein A bead solution thoroughly. For 0.5 μg of antibody, pipette 5 μL of beads into a new set of ChIP 8-tube strips and pulse spin.
5. Fill the last row of the ChIP liquid-handler with labeled, empty ChIP 8-tube strips.
6. Follow the *ChIP-16-IPure-200D* program specifications for the placement of all the strips in the ChIP liquid-handler machine. Add the buffers in the correct position but use tW4 instead of tE1 buffer.
  NOTE: Organize the day such that the ChIP liquid-handler will perform the ChIP overnight. The program will run for about 16 h for 16 samples. This marks the end of Day 1.

### 4. Transposase integration of library adaptors for library preparation

1. Pre-set a thermomixer to 37 °C and 500 rpm. Cool down a magnet for 0.2 mL tube strips on ice.
2. For 16 samples, prepare 440 μL of tagmentation buffer on ice. Pipette 53 μL into a single new 8-tube strip and keep on ice.
3. In a new 0.2 mL 8-tube strip, add 220 μL of cold tC1 buffer and keep on ice. The 8 strip tubes can hold this volume and still be capped.
4. Remove the “IP samples” strip tube from the ChIP liquid-handler machine (row 12) and cap the tubes prior to pulse-spinning. Capture the beads using the magnet for 8-tube strips for 2 min and carefully remove the supernatant.
5. Transfer 25 μL of the tagmentation buffer to the beads with a multi-channel, remove from the magnet, and mix gently until the beads are homogenous (about 5 times up and down with the pipette set to 20 μL).
6. Cap the tubes and place into the pre-heated thermomixer and incubate for 3 min. Increasing the time will decrease the efficiency of the library preparation.
7. Transfer the tubes to a chilled metal rack and add 100 μL chilled tC1 buffer to each sample. Set a multi-channel pipette to 80 μL and mix the sample until the beads are homogenous, stopping the tagmentation reaction.
8. Place the samples back into the ChIP liquid-handler and proceed with the wash procedure *Washing_for_IP-reacts_16_Ipure*. Ensure the washing is performed twice with tC1 buffer and twice with tW4. The elution should be completed as marked by the program layout, with buffer tE1.
9. Decrosslinking of the DNA
  1. Remove the ChIP 8-tube strips in the last row of the ChIP liquid-handler and add 2 μL RNase A to each sample.
  2. Cap the tubes, pulse-spin, gently mix the beads with a multichannel pipette until the mixture is homogenous, and re-cap the tubes.
  3. Incubate the samples in a thermomixer for 30 mins at 37 °C and 900 rpm.
  4. Remove the samples from the thermomixer, add 2 μL of Proteinase K. Follow the same procedure as 4.9.2 after the addition.
  5. Incubate the samples in a thermomixer for 4 h at 55 °C and 1,250 rpm, followed by 65 °C at 1,000 rpm overnight.
    NOTE: This is the end of Day 2.

### 5. Tagmented DNA fragments purification

1. Label sixteen 1.5 mL tubes with the appropriate sample number and add 400 μL of DNA binding buffer from the DNA clean-up kit to each.
2. Remove the 8-tube strips from the thermomixer and pulse-spin the strips to ensure any evaporated product is retained. Place strips on an 8-strip magnet to capture the beads.
3. Transfer 100 μL of decrosslinked DNA into each of the 1.5 mL tubes. Add 100 μL of the DNA binding buffer to the 8-tube strips to wash the beads and then transfer to the appropriate 1.5 mL tube.
4. Vortex for about 10 s and pulse-spin the 1.5 mL tubes.
5. Load the columns with the 600 μL containing the DNA binding buffer and ChIP sample.
6. Spin samples for 20 s at 10,000 *x g* and reload the column with the flow-through. Spin again with the same conditions and discard the flow-through.
7. Wash the columns twice with 200 μL wash buffer (same centrifugation as the previous step) and discard the flow-through.
8. Dry the columns by centrifuging for 2 min at 12,000 *x g*.
9. Transfer the column to a new 1.5 mL collection tube and add 9 μL warm TE Buffer (pre-heated to 55 °C) directly to the column matrix. Allow the column to incubate for 1 min before centrifugation for 1 min at 10,000 *x g*.
10. Transfer the 9 μL of the elute to an appropriate new set of 8-tube strips.
11. Complete the elution again with 8 μL TE Buffer as before. Transfer the elute into the appropriate 8-tube strips (final volume 17 μL per sample) and keep on ice.

### 6. Amplification and size-selection of the purified samples

1. The following steps use qPCR to determine the number of cycles required for optimal amplification (CtD – Ct determination)
2. Prepare the CtD mix for all samples by multiplying the contents of the CtD Mix Buffer by the number of samples.
3. Dispense 3.6 μL of CtD mix into a qPCR plate and add 1.4 μL of tagmented DNA samples (~10 % of the total volume). Perform the following qPCR: 98 °C for 3 min, 72 °C for 5 min, 98 °C for 30 s, 26 cycles of 98 °C for 10 s, 63 °C for 30 s, and 72 °C for 30 s.
4. Prepare the Amp Mix for all samples by multiplying the contents of the AMP Mix Buffer by the number of samples. Dispense 14 μL of tagmented DNA into separate wells of a PCR plate, then add 2.5 μL of two sequencing index primers (25 μM) to each sample (final reaction volume is 50 μL).
5. Mix the samples by multichannel pipette and perform the amplification program used in the CtD with the appropriate number of cycles.
  NOTE: This is a good stopping point as the amplified samples can be stored at −20 °C for a few weeks. However, the purification can be completed on the same day. For 48 samples, the steps 3 – 6.5 were completed with two other separate batches and then amplified in one batch as described below.
6. Perform post-amplification, size selection, and quantification of tagmented DNA as described below. This can usually be completed with 48 samples (can be completed with fewer samples as desired).
  1. Add 90 μL of paramagnetic beads (1:1.8 ratio) into each well, mix, and allow it to incubate at RT for 2 min.
  2. Capture the beads using a plate magnet and discard the supernatant. Wash the beads 3 times with 200 μL of fresh 80% ethanol without disrupting the bead pellet.
  3. Remove any excess ethanol with 20 μL tips after final wash and leave the beads to dry for 10 min or until cracks appear in the bead pellets.
  4. With the plate still on the magnet, add 40 μL of the pre-warmed water to each well. Seal the plate, vortex thoroughly, and briefly pulse-spin the plate.
  5. Capture the beads by placing the plate back on the magnet and transfer the 40 μL elute to a new “sample” Plate. The samples are now purified, and the next steps enrich fragments ranging from 200–1,000 bp.
  6. Optional QC step: Remove 4 μL from the samples and transfer to a QC plate. Add 4 μL water back to the samples. This determines the percentage of large fragments.
  7. Add 22 μL of paramagnetic beads (1:0.55 ratio) to the samples, carefully mix, and incubate at RT for 2 min.
  8. Place on magnet to capture the beads for 5 min and transfer the supernatants to columns 7–12 of the “sample” plate. Remove the plate from the magnet and add 30 μL of beads (final ratio of 1:1.3). Mix carefully and allow it to sit at RT for 2 min.
  9. Capture the beads for 5 min and then discard the supernatant.
  10. Wash all beads 3 times with 200 μL fresh 80 % ethanol as described previously (step 6.6.2 – 6.6.3).
  11. Once the pellets are dry, elute DNA with 8 μL pre-warmed TE buffer to each well, while still on the magnet.
  12. Remove the plate from the magnet, seal, and vortex thoroughly. Allow the plate to incubate for 2 min at RT, pulse-spin, and place the plate back on the magnet for 2 min. Transfer the supernatant to a new plate (Plate 2).
  13. For maximum recovery, repeat elution with an extra 8 μL of pre-warmed TE buffer. Place the samples into the appropriate wells such that each sample has 16 μL of final library.
    NOTE: At the end of this step there should be two plates (one if no QC plate was completed). The QC plate will have the pre-size-selected fragments and the second plate should have 48 wells of final library (16 μL total).

### 7. Quantify the final libraries and QC samples using a fluorescence assay

1. Complete DNA quantification using a fluorescence quantifying assay or a similar method.
2. If QC quantification was completed, determine the percentage of loss of sample that were < 1,000 bp. There should be no more than about 20% loss – if there was more, there could be an issue with the applied bead ratios.
3. Determine the size of the fragments of each sample, preferably using a capillary electrophoresis machine. To calculate the molar concentration use the following equation: [Library concentration (ng/μL) * 10^6^ ]/[660 * Median fragment size (bp)]).
  NOTE: The libraries are ready to be pooled (equimolar amounts) and sequenced following standard next generation sequencing procedures.

## Representative Results

**Figure 1:**
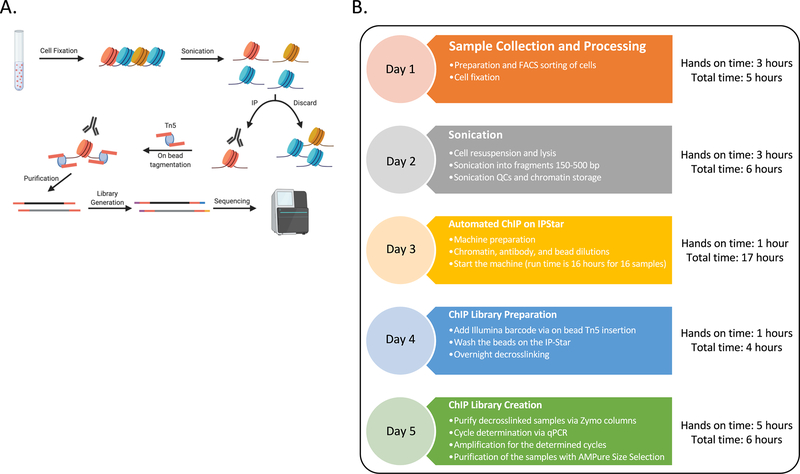
General Flowchart for the procedure. (**A**) A cartoon of the overall procedure (generated in BioRender). (**B**) Flow-chart for all the major steps for the protocol and the estimated hands-on and total time associated with each day. The sequencing could happen at the end of Day 5 or later with multiple rounds. The timeline can also be staggered throughout the week, where sequential Day 3–4 can be completed multiple times in a week to generate 48 ChIP samples.

As proof of concept, ChIP-Seq was completed for six human donors with three sets of immune cell types: naive CD4 T cells (CD4), classical monocytes (MO) and natural killer cells (NK), enriched by FACS sorting as described before^13^. The underlined procedure consists of nine distinct procedures as represented in Figure 1.

**Figure 2:**
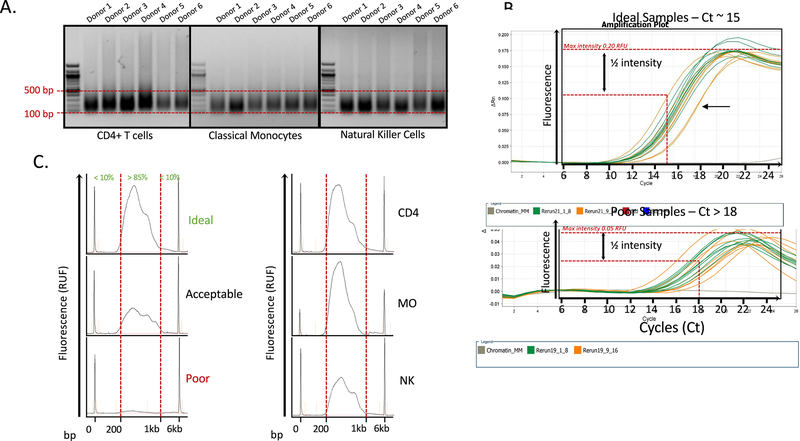
Pre-sequencing QC examples. (**A**) 1.2% agarose gels show reproducibility of sonication. Sonication samples for 6 donors in three cell types: naive CD4 T cells (CD4), Classical monocytes (MO), and Natural killer cells (NK). The samples were sonicated for 14 cycles (16 s ON, 32 s OFF per cycle). For each sample about 200 ng of decrosslinked chromatin were loaded on a 1% agarose gel. Samples were considered good if more than 70 % of the fragments are within 100–500 bp. (**B**) Top - Analysis qPCR amplification curves to determine the optimal number of cycles for amplification (Ct where there is ½ the max intensity). The ideal samples have a Ct of about 15 and amplification can be completed up to 2 cycle more of the measured Ct. The arrow is an example of a bad example where the Ct is greater than 18. Bottom - An example of a poor set of samples is shown which have a Ct greater than 18. These samples also showed lower fluorescence intensity. (**C**) Left - Fragment analyzer electrophoresis traces showed the distribution of final tagmented libraries after amplification and size-selection. Samples with more than 85 % of fragment library lies within 200–1,000 bp were considered as good samples. Measurement of peak intensity of fluorescence is also considered as an important QC parameter, indeed if signal is low, the sample is unlikely to sequence well. Right - Examples for positive samples in CD4, MO, and NK are shown.

After cell isolation by flow cytometry^13^, sorted cells were centrifuged and cells fixed and stored as described above. Once all the samples were collected, the samples were lysed and prepared for chromatic shearing in batches of 12 as described above. For each sample, the number of cycles to reach optimal sonication was completed^10^. Quantitative measurement, as well as sheared chromatin fragment size measurements showed great reproducibility of our method on the three sets of immune cells (Figure 2A). The different human immune cells were sonicated in separate batches and yielded very consistently with > 70% of the sample between 100 – 500 bp for 14 cycles (16 s ON, 32 s OFF per cycle). At this point, samples with large fragments after sonication (< 70% of the sample between 100 – 500 bp) were considered as failed. These samples could either sonicated for 1–2 additional cycles or were discarded and replaced later with cells from another pellet. Our method showed none of the samples required more sonication or were eliminated, suggesting absolute robustness of the procedure.

After quantification, the samples were run on a ChIP liquid-handler with H3K27ac antibodies, followed by tagmentation with Tn5 transposase enzyme. To determine the appropriate number of amplification cycles by qPCR, 10% of tagmented samples were used. For the determination of the number of cycles for the amplification of the samples, we find the cycle at which the intensity of the sample is half the average maximum for cycle determination (Figure 2B). Samples with Ct values of more than 18 did not perform well post sequencing and their Ct value was thus indicative of a failed ChIP sample. These samples generally also yielded a lower amount of DNA after amplification. Samples (100,000 cells input) with a Ct value equal or lesser than 15 were ideal and samples between 15 and 18 were acceptable but less consistent post sequencing. For samples with less than 100,000 input cells, the Ct values were usually found between 15 and 18 but did not need more than 18 cycles to yield enough product for sequencing.

After DNA-tagmented amplification, libraries were purified and size-selected to obtain an ideal size distribution, ranging from 200 to 1,000 bp, for the NextGen sequencing. Size distribution assessment on each of the libraries was completed because best sequencing data were obtained when more than 85% of the DNA fragments ranged between 200 to 1,000 bp (Figure 2C). Notably, as the same quantity of DNA (measured by fluorescence quantification) was loaded, it was noticed the samples with lower fluorescence intensity generally sequenced poorly (Figure 2C).

Post sequencing, standard quality controls based on the ENCODE ChIP-Seq guidelines were applied^5, 14, 15^.

**Figure 3:**
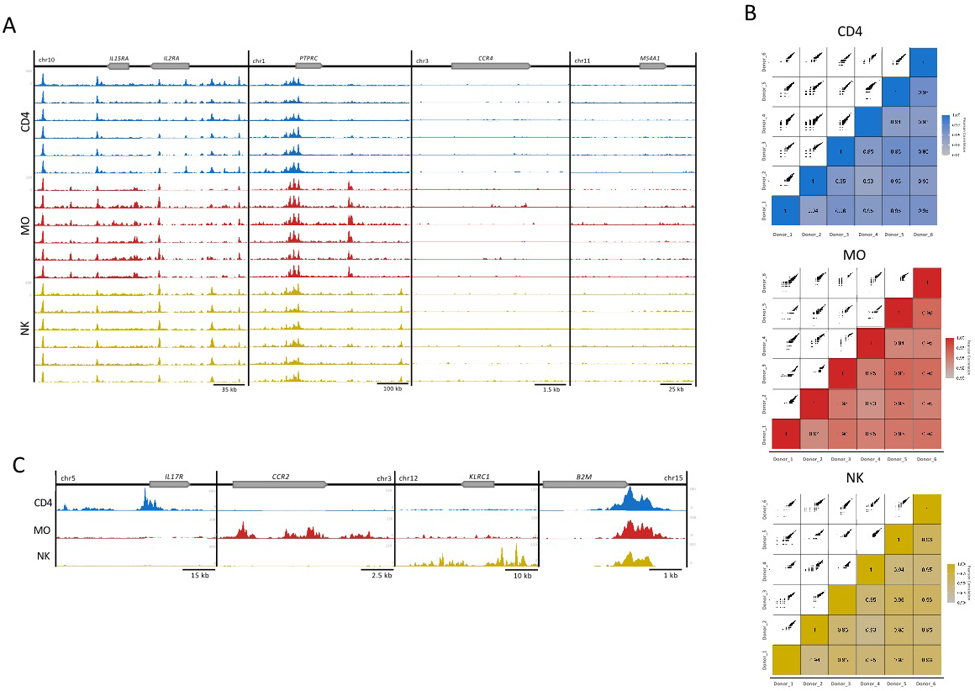
Reproducibility of the immune-cell samples. (**A**) H3K27ac tracks (UCSC Genome Browser, maximum intensity, smoothing function of 4, all with equally scaled Y-axis) for 6 donors (100,000 cells per replicate) in each cell type (CD4, MO, and NK). Four exemplary loci are shown, two with (IL2RA locus and PTPRC) and two without enrichment for H3K27ac (CCR4 and MS4A1). (**B**) Pearson correlation between the donors and corresponding correlation plots generated using a 300 bp extension and 500 bp window within the MEDIPS package for each of the cell type replicates^16^. (**C**) Merged donor files for each cell type showing H3K27ac tracks (UCSC Genome Browser maximum intensity, smoothing function of 4) in cell type-specific regions (IL17R for CD4, CCR2 for MO, and KLRC1 for NK) and the house-keeping gene B2M, present in all cell types.

For visual quality control, H3K27ac enrichment tracks for display in the UCSC genome browser were prepared. For four gene loci, individual tracks for each sample showed high mapping quality and signal-to-noise ratio reflecting the high consistency and robustness of our assay (Figure 3A). The two loci to the left harbor well-expressed genes in these cell types, while the genes in the two loci to the right are not expressed and served as background controls^13^ (Figure 3A). Further, the MEDIPS analysis package was used as post-sequencing variable to assess the correlation index between technical replicates (Figure 3B)^5, 16, 17^, establishing the degree of correlation for reads enrichment level for 500 bp bins^16^. For the majority of the pairwise comparisons, Pearson correlations indexes showed more than 90% correlation suggesting high level of consistence between the biological replicates (Figure 3B). Replicates with acceptable correlation were merged to increase signal-to-noise ratio. While cell type-specific loci showed high enrichment in the appropriate cells, a house-keeping gene (B2M) showed very consistent histone modification (Figure 3C). For the analysis, merging tracks from replicates will increase the enrichment, reinforce the specific signal, including for important cell type-specific enhancers, and reduces the inter-individual variability inherent to human samples^5^.

**Figure 4:**
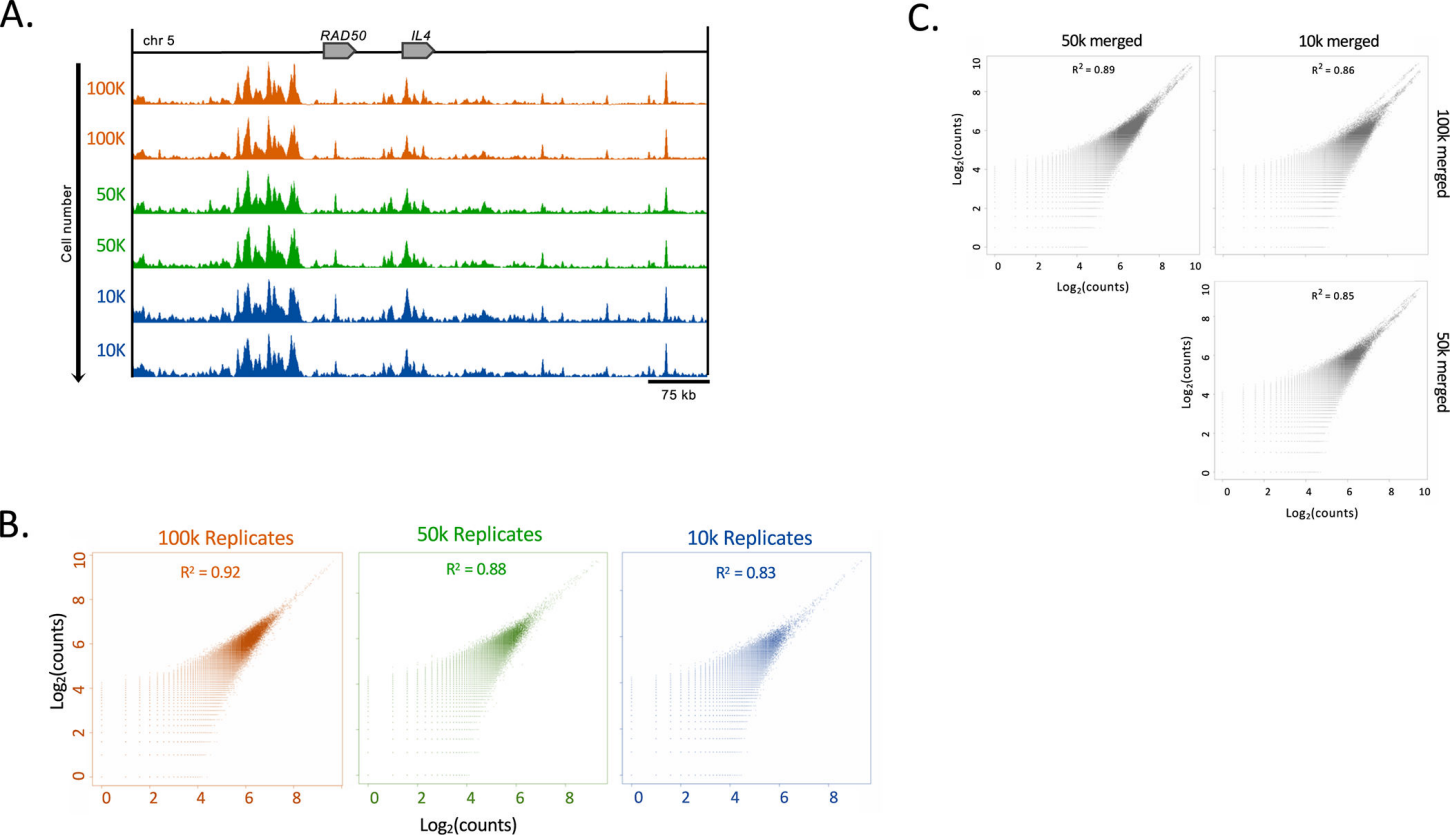
Reproducibility of low input samples. (**A**) Examples of the consistency of H3K27ac ChIP-Seq for cells 100,000 down to 10,000 in HUT-78 cells (a T-cell lymphoma cell line). The tracks (UCSC Genome Browser, maximum intensity, smoothing function of 4, all with equally scaled Y-axis) show the IL4 locus. (**B**) Pearson correlations of the replicates using a 300 bp extension and 500 bp window within the MEDIPS package^16^. (**C**) Pearson correlations between the different cell number groups (100,000, 50,000, and 10,000 cells) using the same MEDIPS parameters as in (B)^16^.

Although 100,000 cells were used for this study, there was high reproducibility for as few as 10,000 cells in a human cultured T-cell line (HUT78). Correlation analysis between ChIP-Seq dataset performed from samples with less than 100,000 cells showed high reproducibility and correlation down to 10,000 cells (Figure 4A).

Pearson correlation analysis showed high correlation index (83% to 92%), suggesting maintenance of signal in low cell number samples. However, there was increased background as the cell numbers were reduced as well as a dropping of the correlation coefficients (Figure 4B). To maintain low background signals, technical duplicates were merged, and the correlation was tested between groups (Figure 4C).

## Discussion

The method described here expands on the ChIPmentation procedure^11^, which implements a tagmentation library preparation protocol prior to DNA purification, by automating and microscaling the protocol. Since the onset of ChIP-Seq, the required cell numbers have been reduced drastically, from about 20 million cells for histones down to hundreds and even single-cells^1, 7, 10, 12, 18, 19, 20, 21^. These newly developed methods have allowed for a deeper understanding of how *cis*-regulatory mechanisms are working in cells by increasing the sensitivity and allowing for rare clinical cell populations to be tested^5, 6, 12, 17^. For instance, one of the more recent and popular procedure, called CUT&TAG, as robust and sensitive ChIP-Seq alternative^9^. It produces an excellent signal-to-noise ratio as the Tn5 enzyme is covalently bound to protein A and recognizes the Fc chain of the ChIP antibody with high specificity^9^. Background activity of Tn5-enzyme is reduced as the enzyme is not functional before binding to the target antibody^9^. However, the implementation of this method in a clinical context is limited since it requires non-fixed, live cells. Also, the removal of DNA fragments from the hypotonic nucleus could have negative effects on the chromatin as it is removed from during the assay. The necessary requirement to work with fresh and living cells is a source of issues for rare clinical samples and for large cohorts of samples, since large cohorts can take numerous years to collect^5^. Another type of method, drop-ChIP, elegantly uses a microfluidics device to generate droplet based tagmention prior to processing the ChIP^19^. However, it uses a highly specialized microfluidic device and, while it is possible to complete single-cell ChIP-Seq, it is also limited to the use of live cells^7, 8, 9, 18, 19^. Newer methods relying on ChIP-Seq such as PLAC-Seq or HiChIP, attempt to understanding 3-dimension (3D) interactions between the ChIP-Seq peaks^22, 23^. These 3D methods are exciting as they are identifying *cis*-regulatory or TF mediated interactions across the genome and better the understanding of the regulation of gene expression in cell types of interest, in healthy tissues and in the context of disease.

There are a few critical steps to consider for the protocol to be successful such as quality of the sonicated chromatin and quality of the antibody. Shearing efficiency is critical, if the chromatin is not sonicated well, the efficiency of the assay decreases drastically^24^. Sonication is a challenging aspect of ChIP-Seq due to the cell numbers required. On the sonicator used in the protocol, efficiency was drastically reduced under 300,000 cells. This is a challenging aspect in ChIP-Seq as to sonicate under that level would often require enzymatic fragmentation, which is less impartial. As a result, sonication is a major limited factor for true microscaled ChIP-Seq. Other sonication platforms and commercially available kits were tested for sonicating chromatin, but the sonicator used here had the most robust and reproducible results. Another advantage of the sonicator is not having to purchase specialized tubes to run the sonication, which reduces costs when dealing with large number of samples. For optimal sonication, firstly, it is important to pre-warm the sonicator as described above. Second, to lyse the pellet, it is recommended to have the pipette tip touching the bottom of the tube while lysing to break up the cells with more physical constrains. Third, any bubble formation prior to sonication hinders the ability of the sample to be sonicated evenly. If there are any bubbles formed during the lysis, it is important to remove them with a pipette. This can be challenging without removing a lot of sample, but if the tip is lightly pressed against the bubble it can be slowly drawn up without loss of much sample. Lastly, when determining the number of cycles, complete a time-course where every three cycles, sample is removed, purified, and ran on an agarose gel. Avoid over/under sonication of samples as this decreases the ChIP efficiency. If the sample is under sonicated, the large fragments can have a negative effect on the ChIP-Seq quality^24^. On the other hand, if the sample is over sonicated, there is a risk of the target epitope getting lost in the process.

Another essential part of ChIP-Seq is the quality of the antibody. Prior to running any large-scale study, it is necessary to optimize the antibody which will be used. The goal is to obtain a significantly high signal to noise ratio of known regions of the genome and another is the reproducibility. If the antibody is pulling a lot of background signal, it might be recommended to use a larger input or try a different lot/supplier. This will add time before starting a large-scale experiment, but it is an essential step. To test for the signal-to-noise it is recommended to use qPCR with regions known to be a target of your antibody and another region known to be absent. It has been noticed histone modifications are more robust and easier to optimize than TFs.

The protocol described above provides a robust method for high-throughput histone-modification ChIP-Seq in a semiautonomous, microscaled manner. The method limits the amount of hands-on time and increases the reproducibility over manual ChIP-Seq. Previous studies completed in the lab used manual ChIP on technical replicates and obtained a Spearman correlation average of 0.50^5^, however, with the semiautomated system, the Spearman correlation between different donors with an NK cells average of 0.66 (Supplementary Table 2). This was also completed with about 40% less hands-on time. The method described here has been optimized for histone-modifications (H3K27ac shown here, but the protocol should not need any modification for others) and would only require minor modifications for to be implemented for TF ChIP-Seq. Despite the quality of the antibody, the main modification would be for the sonication time and potentially the buffers used during the IP. Usually, for TF ChIP assays, the method may work better with slightly longer fragments of chromatin (with a range of around 350–800 bp) as TF:DNA complexes are likely less able to be maintained through rigorous sonication^6^. The buffers might also need to change to a custom mix or other industry available kits, as TFs can behave differently than histone modifications.

Although the automated ChIP liquid-handler was been tested for as few as 10,000 cells, there was a noticeable decrease in reproducibility at lower chromatin concentrations. Due to this, the protocol was not recommended to less than 10,000 cells, with 100,000 cells being the optimal conditions. The protocol was also completed using industry ChIP buffers, which was an added expense but provided higher quality data. The protocol could be modified with regard to the sonication conditions (as long as the sheared chromatin is kept within the same range), buffers could be customized for the immunoprecipitation (IP; optimization may be required), or the ChIP liquid-handler may not be used. A limitation of the protocol is the use of the ChIP liquid-handler, which can be an expensive investment and can only run 16 samples at once. The ChIP liquid-handler is limited to small-scale reactions and cell numbers greater than one million are not recommended. However, the protocol could be completed without it, by completing the IP and wash steps manually. If the IP and washes were completed by hand, the time to complete the assay will increase and the reproducibility may decrease, but this guide will still be useful running a high-quality ChIP-Seq experiment. Of note, other liquid handlers could be adapted to run semiautomated ChIP reactions.

To summarize, the major benefits of this system is the high-throughput nature, since the IP and washing steps are completed autonomously. As such, sequential rounds of ChIP experiments can be completed, allowing up to 48 samples to be fully processed and ready for sequencing in 5 days, with limited hands-on time compared to manual ChIP-Seq experiments. Another benefit is the increased reproducibility since ChIP-Seq can be difficult to obtain highly reproducible results. Other methods either require live cells, complex micro-pipetting systems, or the work to be completed all by hand. This system will have to be optimized for low-input samples (<10,000 cells), ultimately allowing single-cell ChIP reactions. The system is also capable of being adapted for the newer ChIP methods, such as PLAC-Seq and HiChIP^22, 23^.

## Supplementary Material

Supplementary Table 2 Supplementary Table 2: Spearman and Pearson sample correlations for the 6 donors and each cell type.

Supplementary Table 1 Supplementary Table 1: Buffer recipes.

## Figures and Tables

**Table:** 

Name of Material/ Equipment	Company	Catalog Number	Comments/Description	Term in manuscript
AMPure XP for PCR Purification	Beckman Coulter	A63880		SPRI beads
Axygen 0.6 mL MaxyClear Snaplock Microcentrifuge Tube	Corning	MCT-060-C		0.65 mL low binding tube
Bioruptor Pico Sonicator	Diagenode	B01060010	Sonicator used in the lab but others can be used	sonicator
IP-Star Compact Automated System	Diagenode	B03000002	Automated system for ChIP-Seq studies	ChIP liquid-handler
True MicroChIP Kit	Diagenode	C01010130	Contains all the buffers for the IP	ChIP kit
H3K27ac polyclonal antibody - Premium	Diagenode	C15410196		
Tips (bulk)	Diagenode	C30040020	Tips for the IP Star	
200 μl tube strips (8 tubes/strip) + cap strips	Diagenode	C30020002	Strip tubes for use on the IP Star	ChIP 8-tube strip
Medium reagent container for SX-8G IP-Star Compact	Diagenode	C30020003		
Eppendorf ThermoMixer C	Eppendorf	2231000667		
Illumina Tagment DNA Enzyme and Buffer Small Kit	Illumina	20034197		
IDT for Illumina Nextera DNA Unique Dual Indexes	Illumina	20027213		
Proteinase Inhibitor Cocktail	Millipore Sigma	P8340		
Glycine	Millipore Sigma	50046-250G		
Formaldehyde solution	Millipore Sigma	252549-1L		
EGTA pH 8.0	Millipore Sigma	E3889-25G		
Sodium butyrate	Millipore Sigma	303410-100G		
N,N-Dimethylformamide	Millipore Sigma	D4551-250ML	CAUTION - low flash point	
KAPA HiFi HotStart ReadyMix	Roche	KK2601		PCR mix
Proteinase K Solution (20 mg/mL), RNA grade	ThermoFisher	25530049		
PureLink RNase A (20 mg/mL)	ThermoFisher	12091021		
Dynabeads Protein A for Immunoprecipitation	ThermoFisher	10001D		
SYBR Gold Nucleic Acid Gel Stain (10,000X Concentrate in DMSO)	ThermoFisher	S11494		nucleic acid dye
SYBR Green I Nucleic Acid Gel Stain - 10,000X concentrate in DMSO	ThermoFisher	S7563		
Quant-iT PicoGreen dsDNA Reagent	ThermoFisher	P7581	Used in the flourescence quantification	
ROX Reference Dye	ThermoFisher	12223012		
PBS (10X), pH 7.4	ThermoFisher	70011044		
NaCl (5 M), RNase-free	ThermoFisher	AM9760G		
EDTA (0.5 M), pH 8.0, RNase-free	ThermoFisher	AM9260G		
HEPES (1 M) pH 7.5	ThermoFisher	15630080		
UltraPure 1M Tris-HCl, pH 8.0	ThermoFisher	15568025		
UltraPure SDS Solution, 10%	ThermoFisher	24730020		
MgCl2 (magnesium chloride) (25 mM)	ThermoFisher	R0971		
TE Buffer	ThermoFisher	12090015		
QuantStudio 6 Flex Real-Time PCR System	ThermoFisher	4485699		qPCR
PCR Flex-free 8-tube stripes, attached individual optical caps	USA Scientific	1402-4700		8 strip tubes, 0.2 mL 8-tube strip
ChIP DNA Clean & Concentrator (Capped Columns)	Zymo Research	D5205		DNA clean-up kit
